# Intensive Medical Nutrition Therapy Alone or with Added Metformin to Prevent Gestational Diabetes Mellitus among High-Risk Mexican Women: A Randomized Clinical Trial

**DOI:** 10.3390/nu14010062

**Published:** 2021-12-24

**Authors:** Otilia Perichart-Perera, Jennifer Mier-Cabrera, Claudia Montserrat Flores-Robles, Nayeli Martínez-Cruz, Lidia Arce-Sánchez, Itzel Nallely Alvarado-Maldonado, Araceli Montoya-Estrada, José Romo-Yañez, Ameyalli Mariana Rodríguez-Cano, Guadalupe Estrada-Gutierrez, Salvador Espino y Sosa, Mario Guzmán-Huerta, Rodrigo Ayala-Yañez, Enrique Reyes-Muñoz

**Affiliations:** 1 Department of Nutrition and Bioprogramming, Instituto Nacional de Perinatología “Isidro Espinosa de los Reyes”, Montes Urales 800, Mexico City 11000, Mexico; oti_perichart@yahoo.com (O.P.-P.); rocameyalli@gmail.com (A.M.R.-C.); 2 Departamento de Salud, Universidad Iberoamericana, Ciudad de México, Prolongación Paseo de la Reforma 880, Mexico City 01219, Mexico; jennifer.mier@gmail.com (J.M.-C.); nayemc_21@hotmail.com (N.M.-C.); 3 Department of Endocrinology, Instituto Nacional de Perinatología “Isidro Espinosa de los Reyes”, Montes Urales 800, Mexico City 11000, Mexico; cmontsefr@gmail.com (C.M.F.-R.); li_arce@yahoo.com.mx (L.A.-S.); itzel_boo@hotmail.com (I.N.A.-M.); 4 Coordination of Gynecological and Perinatal Endocrinology, Instituto Nacional de Perinatología “Isidro Espinosa de los Reyes”, Montes Urales 800, Mexico City 11000, Mexico; ara_mones@hotmail.com (A.M.-E.); jryz@yahoo.com (J.R.-Y.); 5 Research Direction, Instituto Nacional de Perinatología, “Isidro Espinosa de los Reyes”, Montes Urales 800, Mexico City 11000, Mexico; gpestrad@gmail.com; 6 Clinical Research Branch, Instituto Nacional de Perinatología “Isidro Espinosa de los Reyes”, Montes Urales 800, Mexico City 11000, Mexico; salvadorespino@gmail.com; 7 Department of Translational Medicine, Instituto Nacional de Perinatología “Isidro Espinosa de los Reyes”, Montes Urales 800, Mexico City 11000, Mexico; mguzmanhuerta@yahoo.com.mx; 8 Direction of Centro de Investigación Materno Infantil del Grupo de Estudios al Nacimiento, CIMIGEN, Avenida Tlahuac 1004, Mexico City 09890, Mexico; drayalagineco@gmail.com; 9 Deparment of Gynecologist and Obstetrics, Centro Medico ABC, Avenida Carlos Graef Fernández 154, Mexico City 05300, Mexico

**Keywords:** diet, pregnancy, gestational weight gain, gestational diabetes, obesity

## Abstract

The aim of this study was to examine the efficacy of intensive medical nutrition therapy (MNT) plus metformin in preventing gestational diabetes mellitus (GDM) among high-risk Mexican women. An open-label randomized clinical trial was conducted. Inclusion criteria were pregnant women with three or more GDM risk factors: Latino ethnic group, maternal age >35 years, body mass index >25 kg/m^2^, insulin resistance, and a history of previous GDM, prediabetes, a macrosomic neonate, polycystic ovarian syndrome, or a first-degree relative with type 2 diabetes. Women before 15 weeks of gestation were assigned to group 1 (*n* = 45): intensive MNT-plus metformin (850 mg twice/day) or group 2 (*n* = 45): intensive MNT without metformin. Intensive MNT included individual dietary counseling, with ≤50% of total energy from high carbohydrates. The primary outcome was the GDM incidence according to the International Association of Diabetes Pregnancy Study Groups criteria. There were no significant differences in baseline characteristics and adverse perinatal outcomes between the groups. The GDM incidence was *n* = 11 (24.4%) in the MNT plus metformin group versus *n* = 7 (15.5%) in the MNT without metformin group: *p* = 0.42 (RR: 1.57 [95% CI: 0.67–3.68]). There is no benefit in adding metformin to intensive MNT to prevent GDM among high-risk Mexican women. Clinical trials registration: NCT01675310.

## 1. Introduction

For a long time, gestational diabetes (GDM) was defined as any degree of glucose intolerance that was first recognized during pregnancy. It is now recognized that many of these women detected with hyperglycemia during pregnancy represent preexisting diabetes, so in the present, women found to have diabetes in their first clinical visit by standard diagnosis criteria (fasting plasma glucose >126 mg/dL, 2-h glucose >200 mg/dL during 75 g glucose tolerance test or HbA1c >6.5%) are excluded from GDM definition [1]. According to the International Association of the Diabetes and Pregnancy Study Groups (IADPGS), women with milder degrees of hyperglycemia, such as fasting ≥92 mg/dL, 1-h ≥ 180 mg/dL, and 2-h ≥ 153 mg/dL are diagnosed with GDM. [1]. For women, GDM has been associated with an increased risk of developing preeclampsia [2]. Women with GDM also have a higher risk of developing type 2 diabetes heart disease and suffering from stroke later in life [3]. Babies born to mothers with GDM are at an increased risk of being large for their gestational age, having macrosomia, and having shoulder dystocia [2]. Later in life, these babies are at a higher risk of being overweight and developing type 2 diabetes [3].

The International Diabetes Federation estimated that 20.4 million or 15.8% of live births to women in 2019 had some form of hyperglycemia in pregnancy; the highest prevalence was found in the South-East Asia Region (27.0%), with North America and the Caribbean Region coming in second (21.4%) [4]. A study on Mexican women reported the prevalence to be GDM of 10.3% using the 2010 American Diabetes Association criteria and 30.3% using the International Association of Diabetes and Pregnancy (IADPSG) criteria [5].

High-risk factors for developing pregestational diabetes and GDM include a body mass index (BMI) >25 kg/m^2^ plus one or more of the following additional risk factors: physical inactivity, a first-degree relative with diabetes, a high-risk race or ethnicity (e.g., African American, Latino, Native American, Asian American, Pacific Islander), having previously given birth to an infant weighing 4000 g or more, previous GDM, previous hypertension (140/90 mmHg or having therapy for hypertension), a high-density lipoprotein cholesterol level less than 35 mg/dL, a triglyceride level greater than 250 mg/dL, polycystic ovarian syndrome, glycated hemoglobin ≥5.7%, impaired glucose tolerance or impaired fasting glucose in previous testing, insulin resistance, or a history of cardiovascular disease [1,6].

In the context of a worldwide obesity epidemic, most pregnant women have at least one risk factor for GDM. Effective pre-pregnancy prevention strategies are limited, and the size of the target population is overwhelming [7]. Although several individual trials examining pregnant women in the area of mixed lifestyle interventions have demonstrated significant reductions in the risk of GDM, many more have showed no effect [7].

A systematic review reported that diets such as the Mediterranean diet, Dietary Approaches to Stop Hypertension, and the Alternate Healthy Eating Index were associated with a 15–38% reduced relative risk (RR) of GDM [8]. Likewise, any pre-pregnancy or early pregnancy physical activity was associated with 30% and 21% reduced odds of GDM, respectively [8]. In contrast, frequent consumption of potatoes, meat or processed meats, and protein derived from animal sources was associated with an increased risk of GDM [8]. However, despite extensive research evaluating the effectiveness of lifestyle interventions incorporating diet and/or exercise, there remains a lack of a definitive consensus on their overall efficacy alone or in combination for both the prevention and treatment of GDM [9].

In recent years, metformin has gained acceptance as a safe, effective, and rational option for reducing insulin resistance in pregnant women with type 2 diabetes, GDM or polycystic ovarian syndrome, and it may also provide benefits for obese non-diabetic women during pregnancy [10]. A Cochrane systematic review reported that metformin versus a placebo given to obese pregnant women possibly reduced the risk of GDM (RR: 0.85, 95% CI 0.61–1.19; 3 studies, 892 women; moderate-quality evidence) [11].

Few studies have evaluated the effects that combined interventions have on high-risk women developing GDM. It is unknown if the use of metformin may confer additional benefits in the prevention of GDM when offering intensive medical nutrition therapy (MNT) to women with three or more high-risk factors. Therefore, the aim of this study was to evaluate the efficacy of intensive MNT plus metformin in preventing GDM among high-risk pregnant Mexican women.

## 2. Materials and Methods

### 2.1. Participants

This open-label randomized clinical trial was approved by the Ethics and Research Internal Review Board of the Instituto Nacional de Perinatología in Mexico City (register number: 212250-42131). All participants gave written informed consent. We included pregnant women who received prenatal care in our institution from 1 March 2012 to 31 March 2015. Inclusion criteria were singleton pregnancy before 15 weeks of gestation; a maternal age >18 years; three or more risk factors for GDM: Latino ethnic group, a maternal age >35 years, pregestational BMI > 25 kg/m^2^ [1,6], insulin resistance (determined by the homeostasis model assessment-estimated insulin resistance [HOMA-IR] insulin (μU/mL) × glucose (mg/dL)/405 being >2.5 at admission) [12], and a history of one of the following: GDM in a previous pregnancy, prediabetes [1], a macrosomic neonate (weight > 4000 g), polycystic ovarian syndrome, or a first-degree relative with type 2 diabetes mellitus. Exclusion criteria were multiple pregnancy, contraindication to the use of metformin; ref. [13], women with two or more altered values during 75 g 2-h oral glucose tolerance test (OGTT); fasting: ≥95 mg/dL, 1-h ≥ 180 mg/dL and 2-h ≥ 155 mg/dL before 15 weeks of gestation according to the institutional guidelines [14,15]; any type of pregestational diabetes mellitus; and/or the following pathologies: hyperthyroidism, systemic lupus erythematosus, rheumatoid arthritis, heart disease, chronic hypertension, kidney disease, or liver disease. Elimination criteria were missing two or more scheduled follow-up visits during the prenatal care or a request to abandon the study.

### 2.2. Procedure

All the women were screened for risk factors at the Maternal Fetal Medicine Department during the first trimester ultrasound evaluation. The women who met the inclusion criteria were invited to participate. First, the women were screened for GDM with a 75 g 2-h OGTT before 15 weeks of gestation. After giving written informed consent, the women were randomly assigned to one of two groups: group 1 (intensive MNT plus metformin 850 mg orally every 12 h (Predial^®^, Laboratorios Silanes, S.A. de C.V., Mexico City, Mexico) or group 2 (intensive MNT without metformin). Assigned intervention was done using opaque sealed envelopes containing instructions according to the study group assignment. A detailed medical and obstetric history was recorded. Subsequent visits included prenatal control; obstetric management was carried out by an obstetrician following institutional guidelines. The women received obstetric care every 4 weeks until 32 weeks of gestation, and every 2 weeks from 32 weeks until delivery. In each follow-up consultation, adherence to a diet and metformin use was evaluated and registered in the clinical record. Detection of GDM was performed between 24 and 28 weeks of gestation with a 75 g 2-h OGTT. The women diagnosed with GDM were referred to endocrinology and nutrition consultation for proper control and monitoring every 2 weeks. Glycemic control in women with GDM was defined as achieving 80% or more of self-monitoring of capillary blood glucose in the target value: fasting 70–94 mg/dL and 1 h postprandial <140 mg/dL [16], revised every 2 weeks. GDM management included (a) diet recommendations according to pregestational BMI and weight gain, a carbohydrate restriction of 40–45%, healthy eating recommendations, and diabetes education; (b) self-monitoring of capillary glucose; and (c) for women who did not achieve glycemic control, metformin treatment was started in group 2 (850 mg every 12 h) and doses of metformin were increased to 850 mg every 8 h in group 1. If needed, additional insulin was prescribed until glycemic control was achieved. From randomized allocation until GDM diagnosis, none of the women received treatment with insulin (group 1 or 2) or metformin (group 2) until the primary outcome was assessed (OGTT at 24–28 weeks of gestation).

### 2.3. Intensive Medical Nutrition Therapy

Intensive MNT was started between 10 and 15 weeks of gestation; it was offered by a clinical nutritionist and included nutrition assessment and individual nutrition counseling and education every month. Pregestational weight was self-reported and BMI was calculated. Obesity classification was determined according to the World Health Organization criteria. Maternal weight gain was evaluated according to the 2009 Institute of Medicine guidelines [17]. Energy requirements were estimated as 30 kcal/kg of the current weight of normal weight women (BMI < 25) and 24 kcal/kg of the current weight in overweight women (pregestational BMI between 25 and 29.9). For women with obesity, the minimum amount of energy prescribed was 1500 kcal/day. Macronutrient recommendations included ≤50% of total energy from carbohydrates, 20–25% from proteins, and 30–35% from fat, with <7% from saturated fat. Nutrition goals were to limit gestational weight gain to the recommended ranges and to promote a healthy dietary pattern during pregnancy. An individual food plan was prescribed following the described energy and nutrient requirements to promote an increase in the intake of fruits, vegetables, low-fat dairy, legumes, whole grains, oily fish, and food sources of monounsaturated fatty acids (avocados, olive and canola oils, etc.). To improve food behaviors and promote adherence to nutrition recommendations, a goal setting approach and portion control strategies were used in each visit. Education themes included healthy eating in pregnancy, food groups, healthy carbohydrates, healthy fats, carbohydrate counting, portion size estimation, and improving food choices. During each visit, the women reported their overall adherence to intensive MNT recommendations on a scale of 0–100%. Dietary assessment was performed using a multiple pass 24-h recall at baseline, and dietary adherence was evaluated using the mean intake from two multiple pass 24-h recalls in the last two visits. The Food Processor SQL software (version 10.4, 2008; ESHA Research, Salem, OR, USA), which included Mexican foods, was used for nutrient analysis. Missing foods were added using the Mexican Food Exchange System or food labels.

### 2.4. Primary Endpoint

The primary outcome was the incidence of GDM in both groups measured at 24–28 weeks of gestation using a 75 g 2-h OGTT. GDM was defined as one or more altered glucose values; fasting was ≥92 mg/dL, 1-h ≥ 180 mg/dL and 2-h ≥ 153 mg/dL, according to the diagnostic criteria stipulated in the recommendations of the IADPSG [18]. According to the institutional guidelines [14,15], only women with two or more abnormal glucose values in the OGTT received specific treatment for GDM during the study period.

### 2.5. Secondary Outcomes

The secondary outcomes included the adverse perinatal or neonatal outcomes described as follows (1) Preeclampsia was defined by systolic blood pressure of 140 mmHg or more or diastolic blood pressure of 90 mmHg or more on two occasions at least 4 h apart after 20 weeks of gestation in a woman with a previously normal blood pressure and proteinuria (300 mg or more per 24 h using urine collection or dipstick reading ≥1+). In the absence of proteinuria, preeclampsia was considered with the presence of thrombocytopenia <100,000/µL, creatinine >1.1 mg/dL, elevated transaminases 2 times above the normal value, pulmonary edema, and/or cerebral/visual symptoms [19]. (2) Gestational hypertension was defined by systolic blood pressure of 140 mmHg or more or diastolic blood pressure of 90 mmHg or more without proteinuria or the aforementioned systemic findings [19]. (3) Preterm birth was defined as a live birth before 37 weeks of gestation [20]. (4) Polyhydramnios was defined by the single deepest pocket of fluid being greater than 8 cm in depth and at least 1 cm in width after 28 weeks of gestation [21]. (5) Oligohydramnios was defined by the single deepest pocket of fluid being smaller than 2 cm in depth and 1 cm in width [21]. (6) Being small for the gestational age (SGA) or large for the gestational age (LGA), was defined as a birth weight below the 10th percentile or above the 90th percentile, respectively, for gestational age and sex-specific birth weight references for the Mexican population [22]. Data of secondary outcomes were obtained from clinical records.

Additionally, during each visit, participants recalled if they perceived any adverse effects associated with the use of metformin, such as headaches, heartburn, dyspepsia, diarrhea, or constipation. An adverse effect was reported as positive if it occurred for 3 or more weeks during the study period.

### 2.6. Sample Size

To test the hypothesis that intensive MNT plus metformin decreases GDM incidence from 30% to 5% in high-risk women [23] with a power of 90% and an alpha error of 0.05, 44 participants per group were required

### 2.7. Statistical Analysis

Statistical analysis was performed according to the CONSORT 2010 statement recommended guidelines for reporting parallel-group randomized trials [24]. Qualitative variables were described using frequencies and percentages, and quantitative variables were described using mean and standard deviation. The chi-square test was used for differences of proportions, and the Student’s *t*-test or Mann–Whitney U test was used for mean differences. RR was calculated using 2 × 2 contingency tables with a 95% confidence interval (95% CI). Statistical analysis was performed using SPSS version 24 (Chicago, IL, USA).

## 3. Results

### 3.1. Women Included in the Study

During the study period, 11,079 pregnant women were attended to at our institution. Of this total, 2220 (20%) women received antenatal care before 15 weeks of gestation and were assessed for eligibility; 144 were eligible and had an initial OGTT performed. Of this total, 13 women were excluded because had an abnormal OGTT. The baseline characteristics and risk factors of 110 eligible pregnant women who declined participation versus 131 women eligible with normal OGTT are shown in Table 1; there were no significant differences between the groups. In Table 2 are shown the baseline characteristics and risk factors of 41 pregnant women who declined enrollment after OGTT versus 90 women who were randomized to the study; there were no significant differences between the groups.

**Figure 1:**
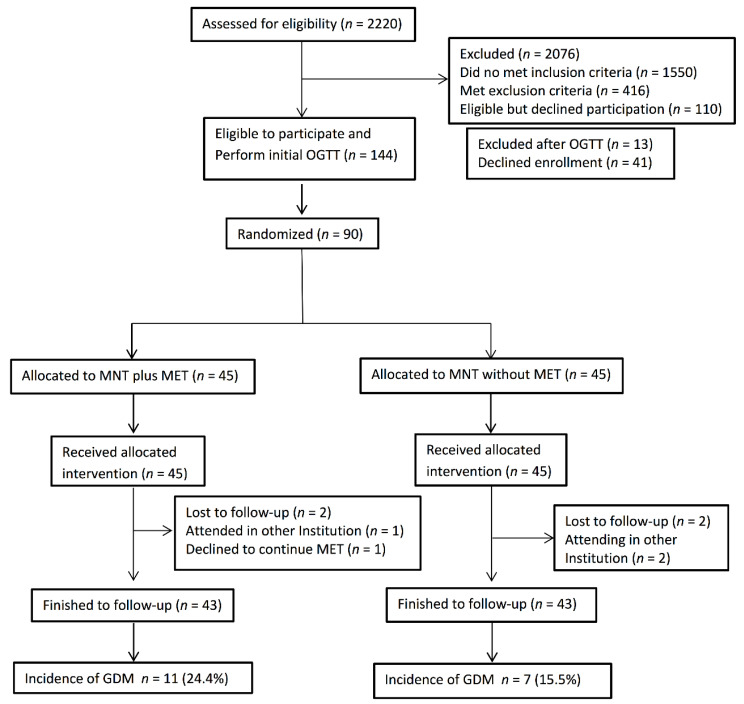
Flow diagram of participants included in the study. OGTT = oral glucose tolerance test MNT = medical nutrition therapy, MET = metformin, GDM = gestational diabetes mellitus.

Figure 1 shows the flow chart of the participants. Forty-five women were included in each. There were 11 cases of GDM in the MNT plus metformin group (24.4%) versus 7 cases in the MNT without metformin group (15.5%); *p* = 0.42 (RR: 1.57, 95% CI: 0.67–3.68). Two women lost during follow-ups in each group.

Of the total number of women diagnosed with GDM, one woman in group 1 required insulin and two women in group 2 required treatment with metformin for glycemic control from 28 to 30 weeks of gestation until delivery.

Baseline characteristics of the two study groups are described in Table 3. There were no significant differences in age, pregestational BMI, weeks of gestation, energy intake, carbohydrate intake, fasting glucose, insulin, HOMA-IR or OGTT values.

Table 4 shows the risk factors and comorbidities of the participants at the beginning of the study. No significant differences were observed between the groups.

### 3.2. Food Intake, Diet Adherence and Gestational Weight Gain

Food intake at admission to the study for groups 1 versus 2 were as follows: total energy, 1922 ± 600 versus 1995 ± 722 kcal/day (*p* = 0.64); carbohydrates, 268 ± 97 versus 279 ± 102 g/day (*p* = 0.62); dietary fiber, 22.1 ± 7.6 versus 25.3 ± 14 g/day (*p* = 0.23); and fat, 28.8 ± 8.5 versus 27.8 ± 6.1% of energy (*p* = 0.57). There were no significant differences between the groups.

Self-reported adherence at the end of the intervention was similar between groups 1 and 2 (85% versus 88%, respectively). No differences were observed in energy, carbohydrates, fiber, or fat intake between the groups (Table 5).

Maternal weight gain was similar in both groups: 9.81 ± 6.2 kg in group 1 and 9.95 ± 4.9 kg in group 2 (*p* = 0.90).

### 3.3. Incidence of Adverse Perinatal Outcomes

Adverse perinatal outcomes are shown in Table 6. There were no significant differences in the incidence of GDM, preeclampsia, gestational hypertension, abnormal amniotic fluid (oligohydramnios or polyhydramnios), preterm delivery, cesarean delivery, or congenital malformations between the two groups.

No significant differences in gestational age at birth, neonatal delivery, newborn weight, and length, LGA or SGA neonates were observed between the groups (Table 7).

### 3.4. Adverse Effects to Metformin

Frequently adverse events associated with the use of metformin in both groups were described in Table 8. No differences between groups were observed.

## 4. Discussion

In the present study, the addition of metformin to intensive MNT did not confer any additional benefits for preventing GDM in women with multiple risk factors.

MNT is defined as a nutrition-based intervention provided by a dietitian nutritionist and is a key component of obesity and diabetes treatment [25]. The intervention usually includes nutrition assessment, dietary counseling, behavioral strategies, education, and monitoring [25]. We offered intensive MNT providing dietary counseling, which limited carbohydrate content (<50%) and promoted a healthy dietary pattern. Portion control and a goal-setting approach were two relevant behavioral strategies used. Education was key, and follow-ups with the women were very frequent (every 4 weeks). A single dietitian offered intensive MNT in both groups, which reduced bias in how the intervention was provided.

Many systematic reviews and meta-analyses have evaluated the effectiveness of different dietary approaches and lifestyle interventions in the prevention of GDM without reporting very promising results [7,9,26]. Most of them concluded that there is a very high heterogeneity among the interventions, among other issues (sample size, baseline risk, GDM diagnostic criteria, methodological issues). It is usually considered a lifestyle intervention when at least one of the following strategies is used, among others: diet, physical activity, education, behavioral change techniques, or self-monitoring [7,9]. The lack of homogeneous intervention groups is an issue in evaluating these types of studies. Inconsistencies in how dietary recommendations and nutrition interventions are delivered have led to confusion in the interpretation of evidence [7].

In the review by Bennett et al. [27], diet, physical activity interventions, and lifestyle interventions that aimed to promote adequate gestational weight gain were evaluated for GDM prevention; diet interventions reduced GDM risk by 44% (RR: 0.56, 95% CI: 0.36–0.87) and physical activity interventions reduced the risk by 38% (RR: 0.62, 95% CI: 0.50–0.78). Lifestyle interventions were not effective except for the Asian population. However, some lifestyle interventions may have included diet counseling; thus, this probably confounded the intervention groups [27].

According to a Cochrane review, there is moderate-quality evidence that combined diet and exercise interventions possibly reduce GDM risk (RR: 0.75, 95% CI: 0.75–1.01, I2 = 42%, *p* = 0.07) [28].

In our study, most women were overweight/obese and the incidence of GDM was high. We did not have a control group without intensive MNT intervention to evaluate the benefit of intensive MNT alone. However, in a study that included participants in our institution with three or more risk factors for GDM without intensive MNT, the incidence of GDM using the IADPSG diagnostic criteria was 46.9% [29], which was higher than the incidence of GDM in this study and could be attributable to the early intervention in the present study.

A recent network meta-analysis of randomized trials was performed to compare the different interventions for the development of GDM in overweight or obese women. None of the interventions were superior to the placebo or no intervention for the prevention of GDM. However, metformin and physical exercise were superior to both the placebo and no intervention for decreasing gestational weight gain [30].

Regarding the effect metformin has on maternal and infant outcomes for pregnant women with obesity or who are overweight, a Cochrane systematic review [11] reported that metformin probably makes little or no difference in the risk of women developing gestational diabetes (RR: 0.85, 95% CI 0.61–1.19). The review concluded that the evidence is insufficient to support the use of metformin for women with obesity in pregnancy for improving maternal and infant outcomes. Likewise, metformin was associated with an increased risk of adverse effects, particularly diarrhea [11]. In the present study the adverse effects were similar in both groups; however, the sample size was limited.

In the EMPOWaR study [31], where obese women (BMI > 30 kg/m^2^) were included and randomized to receive metformin versus a placebo, the incidence of GDM using the IADPSG criteria was 36/153 (24%) in the placebo group versus 26/142 (18%) in the metformin group OR: 0.72 (95% CI: 0.41–1.2, *p* = 0.27). The incidence of GDM was higher than in the present study, which could be attributed to starting intensive MNT before 15 weeks of gestation and receiving nutrition counseling every 4 weeks, as well as the use of the IADPSG criteria. In agreement with this study, our results indicated no benefit from adding metformin, even for women who were obese or overweight and also had multiple risk factors for GDM.

The GRoW study [32] was a multicenter, randomized, double-blind, placebo-controlled trial that evaluated the effects on maternal and infant outcomes of antenatal metformin given in addition to dietary and lifestyle advice among overweight and obese pregnant women. Pregnant women with a BMI of 25 kg/m² or higher were assigned to antenatal dietary and lifestyle intervention plus metformin or a placebo at 10–20 weeks of gestation. The reported incidence of GDM was 27.9% and 23.9% in the metformin and placebo groups, respectively. This is similar to the incidence of GDM in our metformin group; however, the GDM incidence was lower in the MNT group in our study. This could be related to the intensive MNT intervention.

MNT alone in the present study was more effective than MNT plus metformin for preventing GDM. Similar findings were reported in the diabetes prevention program; in nondiabetic persons with elevated fasting and post-load plasma glucose concentrations, the lifestyle intervention reduced the incidence of type 2 diabetes by 58% (95% CI: 48–66%) and treatment with metformin reduced it by 31% (95% CI: 17–43%) compared with the placebo [33].

The strengths of this study were the study design, that most participants had three or more risk factors for GDM, and the early and intensive MNT intervention.

Regarding the limitations of this study, we identified the small sample size and the lack of a control group with the usual prenatal care.

Future randomized clinical trials with a large sample size are necessary to evaluate the effect of early intensive MNT compared to habitual prenatal care on the prevention of GDM in Mexican women.

## 5. Conclusions

There is no benefit in adding metformin to intensive MNT for preventing GDM among high-risk pregnant Mexican women. Intensive MNT that includes counseling about healthy eating and exercise has a role in the prevention of GDM in high-risk Mexican women.

## Figures and Tables

**Table 1:** Baseline characteristics and risk factors of eligible pregnant women who declined participation versus women eligible with initial oral glucose tolerance test (OGTT).

Characteristics	Women Who Declined Participation (*n* = 110)	Women with Initial OGTT (*n* = 131)	*p* Value *
Maternal age (years)	31.9 ± 5	32.3 ± 5	0.59
Pregestational weight (kg)	72.8 ± 10.1	74.0 ± 12.2	0.44
Pregestational BMI (kg/m^2^)	30.0 ± 3.8	30.2± 4.4	0.76
Number of previous gestations	2.6 ± 1.2	2.5 ± 1.3	0.71
Weeks of gestation	13.4 ± 0.8	13.4 ± 1.3	0.99
75 g-2 h OGTT			
Fasting (mg/mL)	-	84.6 ± 8.7	-
1 h (mg/mL)	-	129.5 ± 28.4	-
2 h (mg/mL)	-	119.1 ± 21.2	-
Latino ethnic group	110 (100)	131 (100)	0.98
Maternal age > 35 years	37 (33.6)	47 (35.8)	0.82
Overweight (pBMI 25–29.9 kg/m^2^)	49 (44.5)	56 (42.7)	0.88
Obesity (pBMI ≥ 30 kg/m^2^)	51 (46.4)	65 (49.6)	0.71
Insulin resistance (HOMA-IR > 2.5)	-	66 (50.3)	-
History of GDM	1 (0.9)	1 (0.8)	0.91
History of Macrosomia	4 (3.6)	5 (3.8)	0.76
History of PCOS	12 (10.9)	16 (12.2)	0.70
History of prediabetes	17 (15.4)	22 (16.7)	0.91
History of Infertility	61 (55.5)	74 (56.5)	0.87
First degree relative with DM	70 (63.6)	91 (69.4)	0.41

Values expressed as mean ± standard deviation or frequency and percentage. * Student’s *t*-test or chi-square test. MNT = medical nutrition therapy, Pbmi = pregestational body mass index, HOMA-IR = homeostasis model assessment-estimated insulin resistance, GDM = gestational diabetes mellitus, PCOS = polycystic ovarian syndrome, DM = diabetes mellitus.

**Table 2:** Baseline characteristics and risk factors of pregnant women who declined enrollment after oral glucose tolerance test versus women who were randomized to the study.

Characteristics	Women Who Declined Enrollment (*n* = 41)	Women Randomized at the Study (*n* = 90)	*p* Value *
Maternal age (years)	31.5 ± 6.1	32.6 ± 4.9	0.29
Pregestational weight (kg)	72.7 ± 11.1	74.5 ± 12.7	0.43
Pregestational BMI (kg/m^2^)	30.1 ± 3.9	30.2 ± 4.6	0.81
Number of previous gestations	2.5 ± 1.4	2.4 ± 1.2	0.53
Weeks of gestation	13.4 ± 0.68	13.4 ± 1.5	0.92
75 g-2 h OGTT			
Fasting (mg/mL)	85.8 ± 9.0	83.5 ± 8.2	0.15
1 h (mg/mL)	124.3 ± 32	129.3 ± 26.7	0.34
2 h (mg/mL)	113.6 ± 22.2	114.1 ± 20.8	0.90
Latino ethnic group	41 (100)	90 (100)	0.98
Maternal age > 35 years	14 (34.1)	33 (36.6)	0.53
Overweight (pBMI 25–29.9 kg/m^2^)	17 (41.4)	39 (43.3)	0.99
Obesity (pBMI ≥ 30 kg/m^2^)	21 (51.2)	44 (48.8)	0.95
Insulin resistance (HOMA-IR > 2.5)	19 (46.3)	47 (52.2)	0.66
History of GDM	0 (0)	1 (1.1)	0.57
History of Macrosomia	1 (2.4)	4 (4.4)	0.94
History of PCOS	6 (14.6)	10 (11.1)	0.78
History of prediabetes	6 (14.6)	16 (17.7)	0.84
History of Infertility	25 (60.9)	49 (54.4)	0.61
First degree relative with DM	27 (65.8)	64 (71.1)	0.89

Values expressed as mean ± standard deviation or frequency and percentage. * Student’s *t*-test or chi-square test. MNT = medical nutrition therapy, pBMI = pregestational body mass index, HOMA-IR = homeostasis model assessment-estimated insulin resistance, GDM = gestational diabetes mellitus, PCOS = polycystic ovarian syndrome, DM = diabetes mellitus.

**Table 3:** Baseline characteristics of participants at admission to the study.

Characteristics	Group 1 MNT + Metformin (*n* = 45)	Group 2 MNT (*n* = 45)	*p* Value *
Maternal age (years)	32.4 ± 5.1	32.8 ± 4.7	0.68
Pregestational weight (kg)	73.6 ± 13.9	75.49 ±11.5	0.50
Pregestational BMI (Kg/m^2^)	30.03 ± 5.1	30.45 ± 4.0	0.67
Number of previous gestations	2.3 ± 1.1	2.6 ± 1.5	0.56
Weeks of gestation	13.3 ± 1.5	13.6 ± 1.5	0.40
Total energy intake (Kcal/day)	1922 ± 600	1995 ± 722	0.64
Carbohydrates intake (g/day)	268 ± 97	279 ± 102	0.62
Fasting insulin (μU/mL)	13.9 ± 9.2	13.2 ± 8.5	0.71
HOMA-IR	2.8 ± 1.8	2.7 ± 1.7	0.75
75 g-2 h OGTT			
Fasting (mg/mL)	83.3 ± 8.6	83.7 ± 7.9	0.82
1 h (mg/mL)	129.5 ± 27.6	130.2 ± 25.5	0.90
2 h (mg/mL)	111.3 ± 19.8	117 ± 21.2	0.19

* Student T-test, MNT = medical nutrition therapy, BMI = body mass index, HOMA-IR = homeostasis model assessment-estimated insulin resistance, OGTT = oral glucose tolerance test.

**Table 4:** Risk factors for gestational diabetes mellitus and comorbidities of participants at admission of the study.

Characteristics	Group 1: MNT + Metformin *n* = 45 (%)	Group 2: MNT *n* = 45 (%)	*p* Value *
Risk factors			
Latino ethnic group	45 (100)	45 (100)	0.98
Maternal age > 35 years	17 (37.8)	16 (35.6)	0.83
Overweight (pBMI 25–29.9 kg/m^2^)	18 (40)	21 (46.7)	0.67
Obesity (pBMI ≥ 30 kg/m^2^)	22 (48.9)	22 (48.9)	0.83
Insulin resistance (HOMA >2.5)	26 (57.8)	21 (46.7)	0.29
History of GDM	1 (2.2)	0 (0.0)	0.32
History of Macrosomia	1 (2.2)	3 (6.6)	0.29
History of PCOS	5 (11.1)	5 (11.1)	0.96
History of prediabetes	9 (20)	7 (15.5)	0.54
History of Infertility	21 (46.6)	28 (62.2)	0.13
First degree relative with DM	34 (75.5)	30 (66.6)	0.43
Comorbidities			
Leiomiomas diameter < 3 cm	6 (13.3)	3 (6.7)	0.29
Hypothyroidism	12 (26.6)	14 (31.1)	0.64
Asthma	2 (4.4)	1 (2.2)	0.55
Cervical incompetence	4 (8.9)	4 (8.9)	0.98

* Chi-square test. MNT = medical nutrition therapy, pBMI = pregestational body mass index, HOMA-IR = homeostatic model assessment-estimated insulin resistance, GDM = gestational diabetes mellitus, PCOS = polycystic ovarian syndrome, DM = diabetes mellitus.

**Table 5:** Food intake among the women who received intensive MNT plus metformin versus only intensive MNT to prevent gestational diabetes mellitus at the end of the intervention.

Food Component	Group 1 MNT + Metformin *n* = 45 (%)	Group 2 MNT *n* = 45 (%)	*p* Value *
Energy (Kcal/day)	1804 ± 639	1908 ± 496	0.43
Carbohydrates (g/day)	237 ± 89	260 ±85	0.24
Dietary fiber (g/day)	26.7 ± 11	30 ± 12	0.21
Carbohydrates (%)	52.6 ± 6.6	54.6 ± 8.1	0.23
Fat (%)	29.4 ± 6.3	28.2 ± 7.2	0.43

* Student’s *t*-test. MNT = medical nutrition therapy.

**Table 6:** Relative risk of adverse perinatal outcomes in Mexican women who received intensive MNT plus metformin versus only intensive MNT to prevent gestational diabetes mellitus.

Outcome	Group 1 MNT + Metformin *n* = 45 (%)	Group 2 MNT *n* = 45 (%)	Relative Risk (95% CI)
Gestational diabetes mellitus	11 (24.4)	7 (15.5)	1.57 (0.67–3.68)
Preeclampsia	2 (4.4)	4 (8.8)	0.46 (0.9–2.4)
Gestational hypertension	5 (11.1)	1 (2.2)	5.6 (0.7–44.5)
Polyhydramnios	1 (2.2)	3 (6.7)	0.31 (0.03–2.8)
Oligohydramnios	3 (6.7)	3 (6.7)	0.93 (0.19–4.3)
Preterm birth	5 (11.1)	8 (17.8)	0.58 (0.2–1.6)
Caesarean section	31 (68.9)	35 (77.8)	0.88 (0.7–1.1)
Congenital malformations	0 (0.0)	3 (6.7)	0.25 (0.02–2.1)

MNT = medical nutrition therapy, CI = confidence interval.

**Table 7:** Newborn outcomes of Mexican women who received intensive MNT plus metformin versus only intensive MNT.

Characteristics	Group 1 MNT + Metformin *n* = 43	Group 2 MNT *n* = 43	*p*
Weeks of gestation	37.3 ± 3.8	37.5 ± 2.5	0.71
Length (cm)	48.28 ± 2.6	47.97 ± 3.5	0.65
Weight (g)	2872.8 ± 504	2840.7 ± 556	0.78
Large for gestational age (*n*,%)	1 (2.3)	2 (4.6)	0.52
Small for gestational age (*n*,%)	6 (13.9)	6 (13.9)	0.92
Admission to NICU (*n*,%)	0 (0.0)	1 (2.3)	0.98
Admission to NIMCU (*n*,%)	11 (25.6)	9 (20.9)	0.79
Death (*n*,%)	1 (2.3)	1 (2.3)	0.98

LGA = large for gestational age, SGA = small for gestational age, NICU = neonatal intensive care unit, NIMCU = neonatal intermediate care unit. Values expressed as mean ± standard deviation or frequency and percentage.

**Table 8:** Adverse effects associated with the use of metformin in Mexican women who received intensive MNT plus metformin versus only intensive MNT.

Adverse Effect	Group 1 MNT + Metformin *n*= 45 (%)	Group 2 MNT *n* = 45 (%)	Relative Risk (95% CI)
Headache	3 (6.6)	2 (4.4)	1.5 (0.27–8.7)
Heartburn	15 (33.3)	11 (24.4)	1.4 (0.73–2.6)
Dyspepsia	8 (17.8)	12 (26.7)	0.66 (0.30–1.47)
Diarrhea	2 (4.4)	0 (0.0)	3 (0.32–27.7)
Constipation	5 (11.1)	8 (17.8)	0.64 (0.23–1.8)

MNT = medical nutrition therapy.

## Data Availability

Please contact the corresponding author for data requests.
